# Outcomes of Thoracolumbar Burst Fractures Treated With Open Versus Minimally Invasive Percutaneous Posterior Spinal Stabilization: A Retrospective Study at a Rural Teaching Hospital in South India

**DOI:** 10.7759/cureus.67429

**Published:** 2024-08-21

**Authors:** Gils Thampi, Nagakumar J S, Manoj K Ramachandraiah

**Affiliations:** 1 Department of Orthopaedics, Sri Devaraj Urs Medical College, Sri Devaraj Urs Academy of Higher Education and Research, Kolar, IND

**Keywords:** correction loss, vertebral wedge angle, mcgill pain questionnaire score, vas score, odi score, percutaneous posterior spinal stabilization, thoracolumbar burst fractures

## Abstract

Background

Thoracolumbar spine fractures are the most prevalent type of axial skeleton fractures, with approximately two-thirds occurring between T11 and L2. Percutaneous pedicle screw fixation has been reported to be an effective treatment for thoracolumbar fractures. Minimally invasive percutaneous pedicle screw fixation yields outcomes comparable to those of the standard open procedure and has the advantages of less stress, bleeding, and pain, as well as rapid postoperative recovery. The main objective of this research was to compare the clinical and radiological outcomes of two surgical approaches (open and percutaneous posterior spinal stabilization), concentrating on nonosteoporotic AO Spine Type A3 thoracolumbar burst fractures between T11 and L2.

Materials and methods

We conducted a retrospective study in our hospital, where cases of thoracolumbar burst fractures meeting the inclusion criteria were chosen retrospectively from April 2022 to March 2023. A total of 54 patients (aged 18-60 years) who underwent spinal stabilization were included in this investigation. The population was divided into two cohorts, with 27 patients in each: Group A underwent open posterior spinal stabilization, and Group B underwent percutaneous posterior spinal stabilization. Data retrieved from medical records were analyzed with at least a six-month follow-up, mainly assessing the demographic data, intraoperative parameters, duration of hospitalization, clinical outcomes (Visual Analog Scale, Oswestry Disability Index, and McGill Pain Questionnaire scores), and radiological outcomes (vertebral wedge angle and correction loss).

Results

Both groups had a male preponderance. There were statistically meaningful distinctions between both groups regarding intraoperative parameters (blood loss and surgical duration) and primary clinical outcome parameters (Visual Analog Scale, Oswestry Disability Index, and McGill Pain Questionnaire scores) in the early phase of the study. However, there were no statistically significant differences concerning radiological parameters (vertebral wedge angle and correction loss) or primary clinical outcome parameters at the last follow-up.

Conclusion

The treatment modalities (open and percutaneous posterior spinal stabilization surgery) were equally safe and effective. However, the percutaneous group demonstrated significant reductions in the length of the surgical procedure, blood loss during surgery, duration of hospital stay, and immediate postoperative pain scores, all of which could potentially benefit patients.

## Introduction

Thoracolumbar spine fractures are the most prevalent axial skeleton fractures, with approximately two-thirds of these fractures occurring between T11 and L2 [1]. The AO Spine thoracolumbar classification system categorizes spine injuries of the thoracic and lumbar regions into three main groups, ranked by increasing degree of instability: Types A (compression fractures), B (tension band injuries), and C (dislocations) [2]. Incomplete burst fractures (AO Spine Type A3) comprise most thoracolumbar fractures [3]. While conservative treatment works effectively for some patients with thoracolumbar fractures without neurologic dysfunction, short-segment fixation with pedicle screws has proven to be a more successful technique for stabilizing fractures, correcting kyphosis, and restoring vertebral height [4].

Using a pedicle fixation device with or without arthrodesis, posterior fixation, and stabilization of damaged vertebral segments constitute the standard surgical treatment. The open posterior approach across the midline is used to execute this strategy [5]. Less invasive spinal surgery methods that aim to minimize tissue damage, reduce blood loss, and expedite patient recovery are becoming increasingly popular. The use of percutaneous pedicle screws is a part of minimally invasive surgeries of the spine, and they can be applied in surgical procedures that use a fixation system mainly for the treatment of fractures, degenerative diseases, and tumors [6].

There have been reports of successful treatment of fractures in the thoracolumbar region with percutaneous pedicle screw fixation (PPSF), which is thought to offer the benefits (i.e., alignment restoration and stability of the spinal area) of the open technique without the negative side effects. Minimally invasive PPSF yields outcomes similar to those of standard open surgery and has the advantages of less stress, bleeding, and pain, as well as rapid postoperative recovery [7].

Numerous studies on this topic have concluded that because minimally invasive percutaneous surgeries avoid devascularization of bony structures, extensive retraction forces, and most crucially, denervation of the multifidus and longissimus paraspinal muscle mass, they are significantly more beneficial than traditional open approaches. This translates into faster surgical procedures, lower blood loss, much less postsurgical pain, faster recuperation, and notably, less in-hospital stay [8]. Less reduction force, a loss of recovery height, and simple nut loosening are some drawbacks of percutaneous minimally invasive therapy for thoracolumbar fractures [9].

In this era, where a vast variety of thoracolumbar fracture stabilization techniques exist, it is still controversial whether open or percutaneous stabilization is suitable for a patient. Hence, the goal of this retrospective analysis was to compare mainly clinical and radiological outcomes of two treatment strategies (open and percutaneous posterior spinal stabilization), concentrating on nonosteoporotic AO Spine Type A3 thoracolumbar burst fractures between T11 and L2.

## Materials and methods

A hospital-based retrospective study was conducted among 54 patients who underwent elective open and percutaneous posterior spinal stabilization surgery in R.L. Jalappa Hospital, Tamaka, Kolar, Karnataka, India from April 2022 to March 2023. Ethical clearance was received from the Institutional Ethical Committee of Sri Devaraj Urs Medical College (approval number SDUMC/KLR/IEC/541/2023-24) to conduct the research. Every participant in the research provided written informed consent. In this research, patients aged 18-60 years with one-level burst fractures, specifically AO Type A3, were included. Patients with greater than one-level fractures, osteoporotic/pathological fractures, a history of spinal surgery, mental disease, associated neurological insult requiring posterior canal decompression, or concurrent diseases, including coagulation disorders, stroke, infections, and tumors, were all disregarded in the research.

Patients meeting the inclusion criteria were allocated into two groups: Group A (open group) included patients treated with open posterior spinal stabilization, and Group B (percutaneous group) included patients treated with minimally invasive percutaneous posterior spinal stabilization for thoracolumbar burst fractures. Twenty-seven patients were selected from each group based on a computer-based random number allocation. All patients were operated on by the same surgeon and mobilized the next day of the surgery, irrespective of the study group. Data retrieved from the patients’ medical records were thoroughly analyzed with at least a six-month follow-up. Demographic data, intraoperative parameters (blood loss and surgical duration), and duration of hospital stay were assessed. Oswestry Disability Index (ODI), Visual Analog Scale (VAS), and McGill Pain Questionnaire scores were measured for clinical outcomes, while vertebral wedge angle and correction loss were assessed for radiological outcomes.

Statistical analysis

For evaluation, data were entered into Excel (Microsoft, Redmond, WA, USA) and analyzed using SPSS Statistics standard version 26 (IBM Corp., Armonk, NY, USA). All continuous variables were summarized using means and standard deviations. Categorical variables were summarized using frequencies and proportions. Comparisons of quantitative variables across study groups were performed using independent t-tests, while those of qualitative variables among research groups were done using chi-square tests. A P-value <0.05 was considered statistically significant.

## Results

A total of 54 participants were analyzed. Group A consisted of 27 participants, and Group B comprised 27 participants. The sociodemographic details of the participants are given in Table 1. The mean age of the participants in Group A was 41.51 years, whereas it was 42.96 years for Group B. Both groups were predominantly comprised of male patients.

**Table 1:** Distribution of sociodemographic variables (n = 54)

Variable	Groups	
	Group A (n = 27)	Group B (n = 27)
Age	41.51 ± 13.6	42.96 ± 11.6
Gender		
Male	18 (47.3%)	20 (52.6%)
Female	9 (56.2%)	7 (43.7%)

In the open group, three patients had developed superficial surgical site infections, while in the percutaneous group, no case had developed any complications (Table 2).

**Table 2:** Comparison of the presence of complications (n = 54)

Complication	Group A	Group B	P-value
Yes	3	0	0.298
No	24	27	

The distribution of the VAS, ODI, and McGill Pain Questionnaire scores among the two groups is given in Table 3. On conducting an independent t-test, statistically significant differences in VAS, ODI, and McGill Pain Questionnaire scores were found between both groups for the first week and first-month follow-up. However, at the sixth month, there were no statistically noteworthy differences in VAS, ODI, or McGill Questionnaire scores found between both groups.

**Table 3:** Distribution of Visual Analog Scale (VAS), Oswestry Disability Index (ODI), and McGill Pain Questionnaire scoring among the participants (n = 54)

Time interval		Presurgery		1st week		1st month		6th month	
		Group A	Group B	Group A	Group B	Group A	Group B	Group A	Group B
Scoring									
VAS		8.07 ± 0.26	7.91 ± 0.39	7.07 ± 0.61	4.96 ± 0.7	5.7 ± 0.5	3.96 ± 0.9	3.63 ± 3.5	2.52 ± 0.84
	P-value	0.0820		<0.0001		<0.0001		0.1151	
ODI		69 ± 4.3	69.04 ± 4.1	53.6 ± 3.09	44.5 ± 4.5	40.5 ± 2.6	30.89 ± 3.6	20.9 ± 2.7	19.48 ± 2.8
	P-value	0.97		<0.0001		<0.0001		0.0634	
McGill Pain Questionnaire		63.3 ± 2.2	64 ± 2.8	52.3 ± 2.5	49.2 ± 2.66	45.9 ± 1.8	39.93 ± 2.4	33.07 ± 1.7	32.3 ± 1.6
	P-value	0.3118		0.0001		0.0001		0.0925	

The distribution of hospital stay (days), intraoperative blood loss (ml), and surgical duration (min) among the two groups is given in Table 4. The mean hospital stay for the open group was 11.00 ± 2.112 days. In contrast, the percutaneous group had a notably shorter mean hospital stay of 3.78 ± 1.601 days (P < 0.001). The average intraoperative blood loss for the open group was 358.04 ± 49.934 ml, whereas for the percutaneous group, it was significantly lower at 129.85 ± 32.182 ml (P < 0.001). The mean length of surgery for the open group was 146.81 ± 28.984 minutes, while for the percutaneous group, it was significantly shorter (95.15 ± 13.736 minutes; P < 0.001).

**Table 4:** Comparison of hospital stay, intraoperative blood loss, and surgical duration between the two groups (n = 54)

	Group A	Group B	P-value
Hospital stay (days)	11 ± 2.112	3.78 ± 1.601	<0.001
Intraoperative blood loss (ml)	358.04 ± 49.934	129.85 ± 32.182	<0.001
Surgical duration (min)	146.81 ± 28.984	95.15 ± 13.736	<0.001

Vertebral wedge angle (degrees) comparison between the two groups is given in Table 5. The mean vertebral wedge angle before surgery was 21.04 ± 1.95 degrees for the open group and 21.00 ± 1.80 degrees for the percutaneous group. This difference did not reach statistical significance (P = 0.942). On the first postoperative day, there was no statistically significant difference (P = 0.787) in the mean vertebral wedge angle between the open group (9.85 ± 1.10 degrees) and the percutaneous group (9.78 ± 0.89 degrees). The mean vertebral wedge angle at six months was 12.26 ± 0.98 degrees for the open group and 11.96 ± 0.76 degrees for the percutaneous group. There was no statistically significant difference (P = 0.221).

**Table 5:** Vertebral wedge angle (degree) comparison between the two groups (n = 54)

Time interval	Presurgery		Postsurgery Day 1		6th month	
Radiological parameter (degree)	Group A	Group B	Group A	Group B	Group A	Group B
Vertebral Wedge Angle	21.04 ± 1.95	21.0 ± 1.80	9.85 ± 1.10	9.78 ± 0.89	12.26 ± 0.98	11.96 ± 0.76
P-value	0.942		0.787		0.221	

Correction loss (degree) comparison between the two groups is given in Table 6. At six months, the mean correction loss for the open group was 2.41 ± 0.888 degrees, while for the percutaneous group, it was 2.19 ± 0.834 degrees. There was no statistically significant difference between the groups (P = 0.348).

**Table 6:** Correction loss comparison between the two groups (n = 54)

Time interval	6th month	
Radiological parameter (degree)	Group A	Group B
Correction Loss	2.41 ± 0.888	2.19 ± 0.834
P-value	0.348	

## Discussion

The most frequent spinal injuries resulting from an axial load in which the anterior and middle columns of a vertebral body are affected, with or without flexion force, are thoracolumbar burst fractures [10]. Notably, biomechanical resistance to external force is weak at the T11-L2 level and accounts for a significant fraction of thoracolumbar fractures [11]. The term “three column” was first used in reference to thoracolumbar injuries in 1983 by Denis and McAffee, who also fully redefined the term’s anatomical meaning [12].

Currently, the AO classification system for these injuries is widely accepted due to its ease of use and utility in assisting surgeons in making decisions [13]. This classification indicates that most thoracolumbar fractures are incomplete burst fractures (AO Spine Type A3) [3].

It has been argued that nonoperative management of thoracolumbar burst fractures in the absence of neurologic deficits yields good clinical outcomes; however, the residual kyphotic angle was still notably higher than that of the group that underwent surgical correction. Conversely, in 10%-20% of patients with thoracolumbar burst fractures, nonoperative therapies were linked to late neurologic impairment; these patients subsequently had surgical intervention frequently [14].

Without additional decompression and reduction of spinal fractures, an individual posterior approach may produce favorable clinical effects in individuals with Type A3 spinal fractures [15]. Surgical treatment of thoracolumbar fractures has traditionally been performed mainly using an open midline approach involving fixation of the fracture with a pedicle screw-rod system associated with posterolateral bony fusion [16]. A different approach to treating thoracolumbar fractures is PPSF, which was first utilized to treat degenerative spinal disorders [17]. PPSF was first described by Magerl in 1977 and has become a popular treatment option for thoracolumbar fractures due to the quick development of minimally invasive instruments. Its benefits include reduced intraoperative blood loss, lesser morbidity and perioperative pain, quicker ambulation, and a quicker return to work [18].

Few randomized trials and meta-analyses have been published that demonstrate the superior functional and radiological outcomes of the percutaneous approach over the open approach in the treatment of thoracolumbar fractures. The comparison between traditional open and percutaneous minimally invasive surgeries is still debatable [19].

The chief goal of our retrospective cohort study was to compare clinical and radiological outcomes of two different treatment approaches (open and percutaneous posterior spinal stabilization) for nonosteoporotic AO Spine Type A3 thoracolumbar burst fractures between T11 and L2. Group A (open posterior spinal stabilization) and Group B (minimally invasive percutaneous posterior spinal stabilization) comprised 27 patients each, making a total of 57 patients with thoracolumbar burst fractures with spinal injuries. Demographic and clinical variables, including age and gender, for both groups were not significantly different. This study is analogous to Kumar et al.’s research [20].

We found that the mean intraoperative blood loss for the open group in our study was 358.04 ± 49.934 ml; it was much lower for the percutaneous group (129.85 ± 32.182 ml; P < 0.001), which was statistically significant. Research on the same parameter by Foley et al. [21] and Min et al. [22] similarly revealed a statistically noteworthy decline in intraoperative blood loss in the percutaneous group.

The open group’s average duration of hospital stay was 11.00 ± 2.112 days. Conversely, the group that underwent percutaneous procedures saw a significantly reduced mean hospital stay of 3.78 ± 1.601 days (P < 0.001). According to Khoo et al. [23], patients who received open posterior instrumentation stayed in the hospital for an average of five to seven days, while patients who underwent minimally invasive posterior instrumentation stayed an average of 2.8 days. Wang et al. [24] examined 100 patients who had thoracolumbar fractures and found statistically significant differences favoring the percutaneous group due to shorter hospital stays, less blood lost during surgery, and a faster operating time (all P < 0.05). Similar findings were also found in a meta-analysis of 12 publications that evaluated 279 percutaneous and 340 open operations for thoracolumbar fractures, indicating a mean 5.72-day reduction in hospital stay between the open and percutaneously treated surgical groups [25].

In our investigation, the same surgical team, with sufficient competence in both operations, performed all the surgeries. For the open group, the average operation time was 146.81 ± 28.984 minutes, whereas it was substantially less for the percutaneous group (95.15 ± 13.736 minutes; P < 0.001). According to pooled outcomes, the percutaneous technique had shorter operating times than the open approach in a systematic review and meta-analysis conducted by Phan et al. [25]. When comparing the scores of the VAS, ODI, and McGill Questionnaire between the two groups at one week and one month post-surgery, a statistically significant difference was discovered. However, during the sixth-month follow-up, no notable significant differences were observed in VAS, ODI, or McGill Questionnaire scores between the two groups. Song et al. [26] reported similar findings, wherein the percutaneous group, at three months postoperation, demonstrated a greater improvement in VAS and ODI scores than the open group. As for the VAS and ODI scores, there were no appreciable variations between the two groups at the last follow-up (sixth month). Therefore, the use of the percutaneous technique was linked to early back pain recovery and function, and the degree of paraspinal muscle dissection may have a significant influence on early clinical outcomes.

Upon comparing the radiographic measures, correction loss and vertebral wedge angle, across the study groups prior to the surgery and at the sixth-month follow-up, we discovered no notable significant differences. This outcome is comparable with Lee et al.’s [5] study in terms of the vertebral wedge angle and correction loss.

During the intraoperative and postoperative phases, neither group experienced any significant complications. However, three patients in the open group experienced superficial surgical site infections, which were managed with antibiotics specific to their cultures. In contrast, no complications occurred in any of the patients in the percutaneous group.

Limitations

Our study has certain limitations. A primary constraint of the research is its record-based retrospective methodology. Therefore, new evidence-based understanding of the subject may be provided by prospective randomized controlled trials in the future. However, this disadvantage was lessened because the study was time-bound and the study area had solid documentation processes. Our study’s conclusions cannot be applied to the whole population because it was conducted in a single institution, restricting the wider applicability of the results. Another drawback was the small sample size, which might have prevented reaching firm conclusions.

## Conclusions

Our study focused on comparing both the clinical and radiological outcomes of the open and percutaneous posterior spinal stabilization techniques for AO Type A3 thoracolumbar fractures and did not find any significant differences. Both therapy modalities were equally efficacious and safe. Nonetheless, the percutaneous group demonstrated a noteworthy reduction in the duration of hospitalization, intraoperative blood loss, surgical time, and immediate postoperative pain scores, all of which may be advantageous to such patients.
